# Sound processing in the cricket brain: evidence for a pulse duration filter

**DOI:** 10.1152/jn.00252.2023

**Published:** 2023-09-13

**Authors:** Xinyang Zhang, Berthold Hedwig

**Affiliations:** Department of Zoology, University of Cambridge, Cambridge, United Kingdom

**Keywords:** auditory brain neurons, intracellular recordings, intrinsic oscillations, pulse duration filter, pulse pattern recognition

## Abstract

The auditory system of female crickets allows them to specifically recognize and approach the species-specific male calling song, defined by sound pulses and silent intervals. Auditory brain neurons form a delay-line and coincidence detector network tuned to the pulse period of the male song. We analyzed the impact of changes in pulse duration on the behavior and the responses of the auditory neurons and the network. We confirm that the ascending neuron AN1 and the local neuron LN2 copy the temporal structure of the song. During ongoing long sound pulses, the delay-line neuron LN5 shows additional rebound responses and the coincidence detector neuron LN3 can generate additional bursts of activity, indicating that these may be driven by intrinsic oscillations of the network. Moreover, the response of the feature detector neuron LN4 is shaped by a combination of inhibitory and excitatory synaptic inputs, and LN4 responds even to long sound pulses with a short depolarization and burst of spikes, like to a sound pulse of natural duration. This response property of LN4 indicates a selective auditory pulse duration filter mechanism of the pattern recognition network, which is tuned to the duration of natural pulses. Comparing the tuning of the phonotactic behavior with the tuning of the local auditory brain neurons to the same test patterns, we find no evidence that a modulation of the phonotactic behavior is reflected at the level of the feature detector neurons. This rather suggests that steering to nonattractive pulse patterns is organized at the thoracic level.

**NEW & NOTEWORTHY** Pulse period selectivity has been reported for the cricket delay-line and coincidence detector network, whereas pulse duration selectivity is evident from behavioral tests. Pulses of increasing duration elicit responses in the pattern recognition neurons, which do not parallel the behavioral responses and indicate additional processing mechanisms. Long sound pulses elicit rhythmic rebound activity and additional bursts, whereas the feature detector neuron reveals a pulse duration filter, expanding our understanding of the pattern recognition process.

## Graphical abstract

**Figure:**
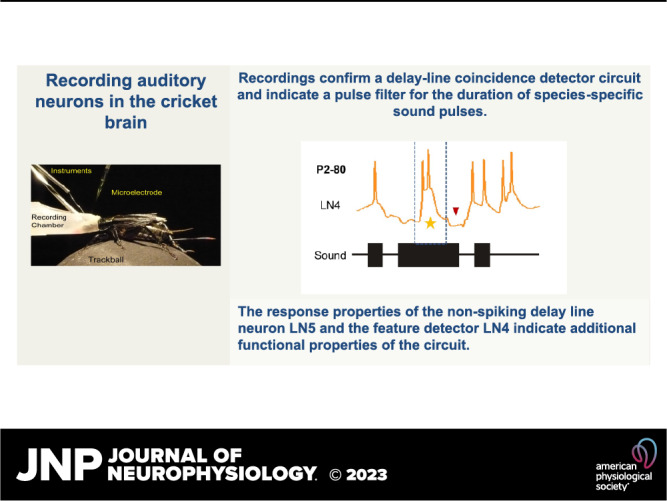

## INTRODUCTION

Akin to Morse code (1), male fireflies and crickets are the most iconic examples to advertise their presence to conspecific females by sequences of constant-frequency light or sound signals, using their bioluminescent lanterns or mechanical stridulatory devices, respectively (2–5). Morse code combines dots, dashes, and corresponding intervals to transmit information, whereas fireflies (*Lampyridae*) (6) and crickets (*Gryllidae*) (7) generally use pulses of constant duration separated by intervals to generate a signal pattern that identifies the species. For example, the calling song pattern of male bispotted field cricket *Gryllus bimaculatus* consists of 17- to 20-ms sound pulses separated by intervals of similar duration, with three to five pulses grouped into chirps separated by a longer interval and repeated at a rate of 2–3 Hz. At the receiver side, i.e., the females, these temporal signal patterns are processed within the central nervous system to allow the selection of conspecific mates.

The phonotactic behavior of female *G. bimaculatus* is narrowly tuned to the temporal characteristics of the male calling song pattern (8, 9). Although orientation of a receiver toward the signal source is supported by low-level parallel neural processing of binaural cues, and frequency processing can be based on the tuning of the afferents, identifying a temporal pattern is more challenging. This requires some form of sequential processing of subsequent pulses within the central nervous system. So far we know very little about the neural processing of flash patterns in fireflies. A more detailed understanding of pattern recognition has been worked out in crickets, narrowing down principal hypotheses on the possible mechanisms underlying song pattern recognition (8, 10, 11). Recordings of auditory brain neurons in the cricket *G. bimaculatus* support the concept of a delay-line and coincidence detection mechanism, in which the neural response to the calling song pulses is forwarded in two separate paths to a coincidence detector (8, 12). As the activity in one of the channels is delayed by the duration of the species-specific pulse period, the coincidence detector will respond best to a sequence of pulses matching the normal song pattern. The details of the neuronal circuitry have been described with the rebound properties of a nonspiking auditory brain neuron providing the delay-line pathway (12). The functional relevance of the network has been supported by a comprehensive modeling study (11), and corresponding neural principles for processing sequences of pulsed signals have been proposed for the mormyrid fish (13, 14) and echo-delay sensitive neurons in bats (15, 16).

Neural responses to sequences of sound pulses separated by silent intervals come with specific consequences for processing: although the pulses initiate excitatory or inhibitory activity reflecting the acoustic pattern, in the silent intervals only the aftermath of these responses can be processed. Therefore, the duration of sound pulses and the duration of the pulse intervals set different constraints for the pattern recognition process (12, 17). The impact of different pulse intervals on the neuronal activity of the pattern recognition network in the brain was described previously (18); here we analyze the impact of systematically changing the duration of individual pulses within a chirp on female phonotactic behavior and the responses of the pattern recognition neurons in the brain of *G. bimaculatus*.

Acoustic stimulation paradigms used are based on behavioral tests by Hedwig and Sarmiento-Ponce (19) demonstrating that variation of a single pulse duration in a chirp of three pulses has a substantial impact on the tuning of female phonotactic behavior. In *G. bimaculatus* pattern recognition is tolerant (20, 21), as females transiently accept unattractive signals when these are preceded by calling song. Our experiments therefore also aimed to reveal where this modulation might occur in the pattern recognition process.

## MATERIALS AND METHODS

### Animals

Adult female crickets (*Gryllus bimaculatus* DeGeer) at 10–25 days postecdysis with intact tympanal membranes and spiracles were used for the experiments. Last-instar nymphs were separated from the colony at the Department of Zoology/Cambridge and kept individually in plastic containers at 28°C with a 12:12-h light-dark cycle and isolated from singing males. Cricket food contained a mixture of muesli, fish food, and cat food, and water was provided ad libitum. All experiments were performed at 23–24°C.

### Acoustic Stimulation

**Figure 1.**
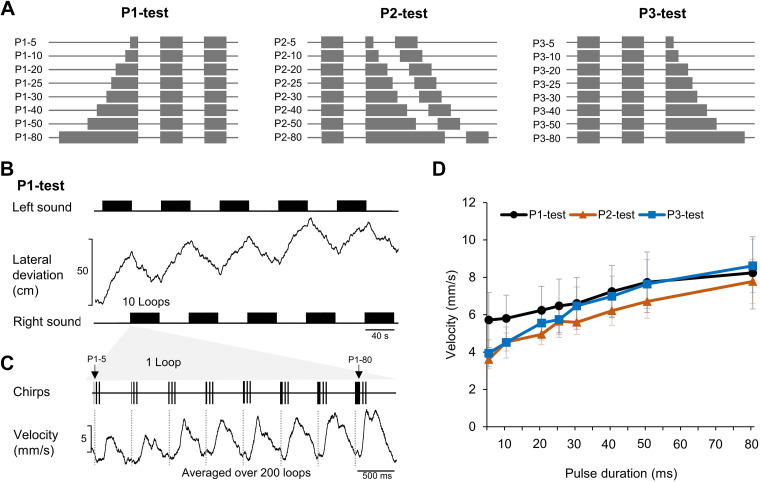
Experimental pulse patterns and behavioral analysis. *A*: test patterns P1-test to P3-test; in each test sequence (loop) the duration of 1 pulse within a 3-pulse chirp is systematically increased from 5 ms to 80 ms while the other pulses and pulse intervals are kept at 20 ms. *B*: steering response as revealed by the lateral deviation of a female cricket to 10 loops of the test paradigm presented from the left or right side. *C*: structure of a single loop (*top*) and averaged steering velocity in response to the chirps of the loop (*bottom*). *D*: steering velocity response to the different test paradigms plotted over the varied pulse duration for the P1-test (black), P2-test (red), and P3-test (blue).

All experiments are based on chirps with three sound pulses (labeled P1 to P3) and two intervals (I1 and I2). Corresponding to behavioral tests (19), three acoustic test paradigms with a carrier frequency of 4.8 kHz were used (Fig. 1*A*). In the P1-test, the first pulse (P1) of chirps was systematically set to 5, 10, 20, 25, 30, 40, 50, or 80 ms, while keeping the duration of all intervals and pulses P2 and P3 at 20 ms (Fig. 1*A*, P1-test). In the P2-test, the second pulse (P2) was systematically adjusted to 5, 10, 20, 25, 30, 40, 50, and 80 ms while the duration of the intervals and pulses P1 and P3 was 20 ms (Fig. 1*A*, P2-test). In the P3-test, the third pulse (P3) was set to 5, 10, 20, 25, 30, 40, 50, and 80 ms while keeping pulses P1 and P2 and the intervals at 20 ms (Fig. 1*A*, P3-test); with different pulse lengths, the chirp duration slightly changed. The chirp period was kept at 500 ms, and chirp patterns were presented in a fixed sequence. The rising and falling ramps for sound pulses were 2 ms. Chirps with pulses and intervals of 20 ms are referred to as “normal chirps,” as they correspond to natural chirps of *G. bimaculatus* (22). Sound stimuli were designed with Cool Edit Pro 2000 software (Syntrillium, Phoenix, AZ). Sound signals were presented by two speakers (Sinus live NEO 13 s; Conrad Electronics, Hirschau, Germany) placed frontal to the cricket at an angle of 45° to the left and the right to the animal’s long axis. Sound intensity was calibrated to 75 dB SPL relative to 20 μPa at the location of the cricket with a Brüel & Kjær measuring amplifier and a 1/2-in. free-field microphone (models 2610 and 4939, respectively; Nærum, Denmark).

### Behavioral Tests

For the P1-, P2-, and P3-tests, all eight chirps with different test pulse durations were combined in one “loop” lasting 4 s; sequences of 10 loops lasting 40 s in total were presented alternatingly from the left and right sides (Fig. 1, *A–C*). This was repeated 10 times, so that each chirp pattern was presented 100 times from the left-hand and 100 times from the right-hand side. Alternating stimulus paradigms were used to eliminate any lateral bias the animals may have when responding to the acoustic test patterns. To reveal the tuning of the auditory steering behavior, we averaged the lateral steering velocity to chirps of the same type as a measure of the auditory evoked motor response (23). We measured for the P1-, P2-, and P3-tests the behavioral responses of 11, 16, and 14 animals, respectively, and pooled the data for the presentation of sound patterns from the left and right sides; the same setup was used as in Ref. 19. We used the looped presentation of the chirp patterns as described previously (18) to analyze the responses of auditory neurons and to compare these with the behavioral data.

### Intracellular Recordings, Staining of the Neurons, and Data Analysis

The same methods were used here for intracellular recordings, staining of the neurons, and data analysis as described previously (18).

For each neuron type a different number of recordings (*N*) and a different number of stimulus repeats (*n*) were obtained. For the characteristic responses of the neurons elicited by 20-ms pulses and normal chirps, 10 repeats of AN1 (*N* = 5, *n* = 10), 10 repeats of LN2 (*N* = 2, *n* = 10), 10 repeats of LN5 (*N* = 4, *n* = 10), 10 repeats of LN3 (*N* = 5, *n* = 10), and 5 repeats of LN4 (*N* = 5, *n* = 5) were analyzed. For the P1-, P2-, and P3-tests, 5 repeats of AN1 (*N* = 5, *n* = 5), 2 repeats of LN2 (*N* = 2, *n* = 2), 18 repeats of LN5 (*N* = 4, *n* = 18), 13 repeats of LN3 (*N* = 5, *n* = 13), and 5 repeats of LN4 (*N* = 5, *n* = 5) were analyzed.

We used the same looped presentation of the chirp patterns as in the behavioral tests to reveal the responses of auditory neurons. To analyze the neural responses, the number of action potentials (APs) per chirp generated was calculated for the four spiking neurons of the circuit, AN1, LN2, LN3, and LN4, together with the standard error of the mean.

For the nonspiking neuron LN5, the resting potential was determined by averaging the membrane potential from −50 ms to 0 ms before the stimulus. The difference between the resting potential and the peak of a postinhibitory rebound (PIR) potential was measured and the standard error of the mean calculated for each rebound amplitude. Because of the sequential signal processing in the network and the latencies of the responses, the peak of the rebound in response to the first sound pulse (i.e., PIR1) occurs after the second sound pulse and the peaks of the second and third rebounds occur after the third sound pulse (i.e., PIR2 and PIR3). Therefore, the latency to the peak of PIR1 is measured in relation to the second pulse and the latency of the subsequent PIRs is measured relative to the third pulse.

Neural responses to test patterns were analyzed by repeated-measures ANOVA with Tukey post hoc test if the data were parametric and Friedman test with Dunn’s multiple comparisons test if the data were nonparametric. Results with a *P* value < 0.05 are considered significant. Pearson’s correlation coefficient was calculated to correlate the amplitude of the postinhibitory rebound and the following inhibition.

## RESULTS

### Behavioral Responses

When presented with looped chirps of the P1-, P2-, or P3-test patterns, female crickets showed clear steering responses toward the acoustic signal, as shown in the example of a P1-test (Fig. 1*B*). The lateral deviation does not directly reveal the specific steering responses to the eight different types of chirps combined in a loop. For each animal we therefore averaged the steering velocity over the time course of a loop for the 200 loops presented in the P1-, P2-, and P3-tests and measured the amplitude of the change in steering velocity initiated by each type of chirp as an indicator of the phonotactic response (Fig. 1*C*) (18). By pooling the data over all females tested (P1-test: *N* = 11; P2-test: *N* = 16; P3-test: *N* = 14), we plotted the tuning curves of the steering response to the changes of the pulse duration. In all three tests, the steering responses continuously increased with the increase of the pulse duration and the tuning curves did not reveal a preference for a particular pulse duration (Fig. 1*D*). These tuning curves were used as reference for the neuronal data. Note that, different from these experiments, the behavior is narrowly tuned to the species-specific temporal pattern if all pulses of a chirp have the same duration and if the patterns are not presented in a looped mode (8, 9, 17).

### Neuronal Activity

**Figure 2.**
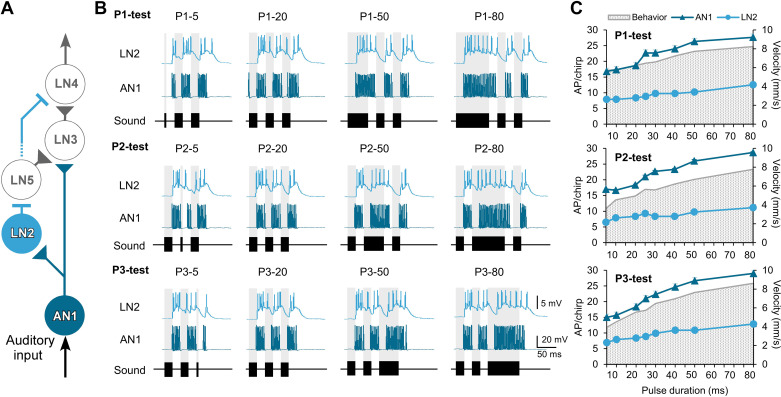
*A*: neural circuit proposed for the delay-line coincidence detector network from Ref. 12, with the neurons AN1 and LN2 highlighted. Excitatory connections are indicated by triangles and inhibitory connections by bars. Note that LN2 also connects to LN4, as indicated by the dotted line. *B*: intracellular recorded responses of AN1 and LN2 to the P1-test (*top*), P2-test (*middle*), and P3-test (*bottom*) for 4 representative chirp patterns, respectively. The recordings of AN1 and LN2 are not performed simultaneously. *C*: tuning of the spiking response of AN1 (dark blue) and LN2 (light blue) in comparison to the behavioral response (gray stippled background). AP, action potential.

The activity of auditory neurons AN1, LN2, LN3, LN4, and LN5 described for the delay-line and coincidence detector circuit in the ventral protocerebrum [e.g., Fig. 2*A*; Schöneich et al. (12)] was recorded intracellularly in response to chirps with systematically varied pulse duration. The responses of the neurons to the normal chirp are included in Supplemental Table S1 and have been described previously (18).

#### Response of the ascending neuron AN1 and of the local neuron LN2.

Spike activity of the ascending neuron AN1 is driven by the afferent activity. It copies and forwards the temporal pattern of 4.8-kHz sound stimuli to the inhibitory local neuron LN2 in the brain (12, 17, 24, 25) (Fig. 2, *A* and *B*). Both neurons responded to the chirps with high fidelity in copying the pulse duration even when the pulse duration was as long as 80 ms. The tuning curves of AN1 in all three tests exhibit an increase with increasing pulse duration (Fig. 2*C*, Table 1), which is in accordance with the tuning of the behavioral activity. The tuning curve of LN2 only showed a slight increase over the duration of the test pulses, and compared to the spike activity elicited in AN1, LN2 showed 58.19 ± 1.11% (P1-test), 59.38 ± 1.47% (P2-test), and 55.17 ± 0.95% (P3-test) fewer spikes, respectively, in response to each test chirp. With their activity patterns, AN1 and LN2 tuning curves followed the tuning of the behavior.

**Table 1.** Number of spikes of the four spiking neurons and the slope of the nonspiking neuron in response to the P1-, P2-, and P3-tests

Chirp	AN1, AP/chirp	LN2, AP/chirp	LN3, AP/chirp	LN4, AP/chirp	LN5 slope
P1-5	16.8 ± 0.3	7.9	6.9 ± 0.6	2.2 ± 0.2	0.045
P1-10	17.3 ± 0.3	7.9	7.6 ± 0.6	3.0 ± 0.3	0.045
P1-20	18.7 ± 0.3	8.4	9.6 ± 0.0	3.8 ± 0.2	0.052
P1-25	22.7 ± 0.7	8.8	10.0 ± 0.4	3.8 ± 0.3	0.048
P1-30	22.7 ± 0.3	9.8	10.2 ± 0.6	3.4 ± 0.5	0.040
P1-40	24.0 ± 0.5	9.8	11.3 ± 0.5	4.0 ± 0.4	0.040
P1-50	26.3 ± 0.3	10.2	10.6 ± 0.4	4.2 ± 0.5	0.027
P1-80	27.7 ± 0.3	12.6	11.6 ± 0.5	3.2 ± 0.4	0.018
P2-5	17.0 ± 0.0	6.5	8.3 ± 0.3	2.2 ± 0.3	0.051
P2-10	16.7 ± 0.3	7.9	8.0 ± 0.6	2.8 ± 0.3	0.045
P2-20	18.3 ± 0.3	8.4	10.3 ± 0.0	3.8 ± 0.3	0.036
P2-25	21.0 ± 0.5	9.3	10.0 ± 0.3	3.2 ± 0.4	0.034
P2-30	22.7 ± 0.3	8.4	10.4 ± 0.2	3.4 ± 0.4	0.030
P2-40	23.3 ± 0.3	8.4	10.3 ± 0.4	3.6 ± 0.5	0.024
P2-50	26.0 ± 0.5	9.8	10.7 ± 0.4	3.8 ± 0.4	0.016
P2-80	28.7 ± 0.3	11.2	12.4 ± 0.4	3.4 ± 0.8	0.016
P3-5	15.0 ± 0.0	6.9	8.0 ± 0.3	2.4 ± 0.2	0.037
P3-10	15.7 ± 0.3	7.9	9.4 ± 0.3	3.0 ± 0.3	0.036
P3-20	18.3 ± 0.3	8.4	9.7 ± 0.0	4.4 ± 0.5	0.038
P3-25	21.0 ± 0.0	8.9	10.1 ± 0.2	4.0 ± 0.3	0.034
P3-30	22.3 ± 0.3	9.9	10.0 ± 0.3	4.0 ± 0.3	0.032
P3-40	24.7 ± 0.3	10.9	10.2 ± 0.4	3.8 ± 0.2	0.034
P3-50	26.7 ± 0.3	10.9	10.4 ± 0.5	3.8 ± 0.2	0.034
P3-80	29.0 ± 0.0	12.9	12.0 ± 0.6	3.6 ± 0.2	0.033

Responses are given with SE. AP, action potentials.

#### Response dynamics of the nonspiking delay-line neuron LN5.

**Figure 3.**
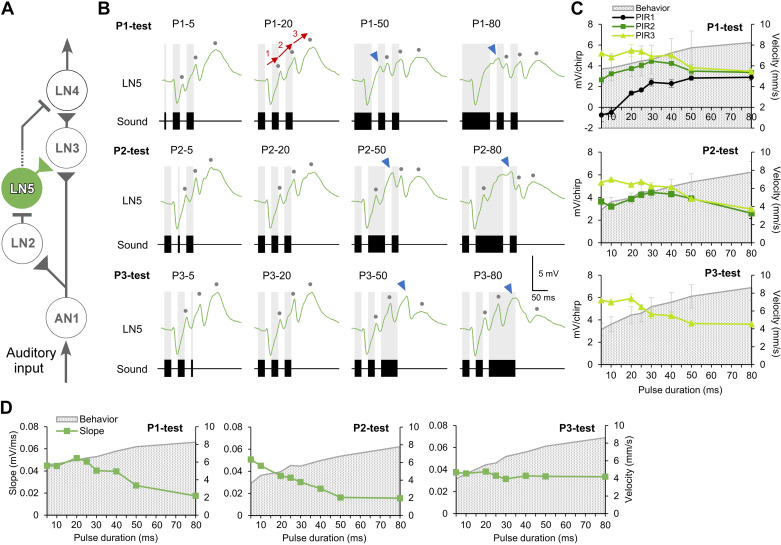
*A*: the proposed pattern recognition circuit with the delay-line neuron LN5 highlighted. *B*: intracellular recorded responses of LN5 to the P1-test (*top*), P2-test (*middle*), and P3-test (*bottom*) for 4 representative chirp patterns, respectively. Note the sequential occurrence of inhibition and postinhibitory rebounds. Gray dots indicate the postinhibitory rebounds PIR1 to PIR3 following the inhibition in response to the 3 sound pulses of a chirp, respectively. Blue arrowheads indicate additional rebounds in response to long sound pulses. Red arrows for P1-20 indicate the increase of the rebound potential over the time course of a chirp, used for calculating the slope of the membrane potential change. *C*: tuning of the different postinhibitory rebounds PIR1 (black), PIR2 (dark green), and PIR3 (light green) in comparison to the behavioral response (gray stippled background). *D*: comparison of the overall increase of the LN5 rebound potential (slope, see P1-20) with the behavioral tuning for the 3 pulse patterns tested.

The nonspiking brain neuron LN5 plays a key role in generating a delayed rebound excitation matching the timing of the species-specific pulse pattern (12). Its membrane potential is driven by the inhibitory input from LN2 (Fig. 3*A*) and by its intrinsic properties. Normal chirps elicited a continuously rising membrane potential oscillation driven by the pulse pattern with a typical sequence of inhibition and postinhibitory rebound depolarization (Fig. 3*B*, P1-20, P2-20, P3-20; see Ref. 18). All normal chirps in the three tests elicited three distinct postinhibitory rebounds (Fig. 3*B*, gray dots). For long P1 test pulses (P1-40, P1-50, and P1-80), the rebound had more time to develop and the amplitude of the first rebound was larger than those elicited by shorter P1s (Fig. 3*B*). The peak of the last rebound decreased to almost the same level as the first and the second rebound in response to P1-50 and P1-80; thus the difference between the second and the third rebound became less prominent when P1 increased in duration.

The responses of LN5 to P2- and P3-tests were similar as in the P1-test: extended sound pulses led to a more pronounced rebound (Fig. 3*B*). Overall, in response to chirps with P1, P2, or P3 longer than 30 ms, a deflection of the membrane potential occurred in the rising phase of the rebound. For longer pulses of 50 and 80 ms the deflection developed into an extra peak that exceeded the amplitude of the corresponding postinhibitory rebound (Fig. 3*B*, blue arrowheads). The extra peak occurred with a constant delay to the offset of the corresponding sound pulses in P1- and P2-tests (P1: 29.9 ± 0.5 ms; P2: 28.3 ± 0.2 ms), whereas the delay was longer in the P3-test (P3: 35.5 ± 0.4 ms) (Table 2).

**Table 2.** Timing of the extra depolarization (bump) occurring in the postinhibitory rebound of LN5

After the Offset of P1		After the Offset of P2		After the Offset of P3	
P1-25	31.6	P2-25	28.2	P3-25	35.6
P1-30	30.2	P2-30	28.5	P3-30	34.8
P1-40	29.9	P2-40	27.9	P3-40	35.8
P1-50	29.4	P2-50	27.8	P3-50	34.4
P1-80	28.2	P2-80	28.9	P3-80	37.1
Average	29.9 ± 0.5	Average	28.3 ± 0.2	Average	35.5 ± 0.4

Values are given in milliseconds.

For a quantitative analysis of LN5 activity, the amplitudes of the PIRs following the test pulse in all three tests were calculated and plotted: PIR1, PIR2, and PIR3 in the P1-test; PIR2 and PIR3 in the P2-test; PIR3 in the P3-test (Table 3, Fig. 3*C*). Among the tuning curves of the three tests, only the tuning of PIR1 in the P1-test showed a continuous increase, matching the tuning of the phonotactic response; the tuning curve of PIR1 increased linearly from −0.7 ± 0.7 to 2.4 ± 0.3 mV (m = 0.1322, *R*^2^ = 0.9768, correlation coefficient) when the P1-pulse increased from 5 to 30 ms; it then increased more gradually from 2.4 ± 0.3 to 2.9 ± 0.3 mV (m = 0.0113, *R*^2^ = 0.6686, correlation coefficient) for P1-30 to P1-80. The tuning curves of all other PIRs in the P1-, P2-, and P3-tests showed trends of decreasing amplitudes with increasing pulse duration and did not reflect the phonotactic response.

**Table 3.** Amplitude of the PIRs of LN5 in response to the three test patterns

Chirp	PIR1	PIR2	PIR3
P1-5	−0.7 ± 0.6	2.7 ± 0.3	5.2 ± 0.3
P1-10	−0.5 ± 0.3	3.2 ± 0.1	4.8 ± 0.4
P1-20	1.3 ± 0.2	3.7 ± 0.1	5.5 ± 0.6
P1-25	1.7 ± 0.1	4.0 ± 0.3	5.4 ± 0.2
P1-30	2.4 ± 0.3	4.5 ± 0.2	4.9 ± 0.4
P1-40	2.3 ± 0.4	4.2 ± 0.2	5.0 ± 0.3
P1-50	2.8 ± 0.2	3.5 ± 0.3	3.8 ± 0.2
P1-80	2.9 ± 0.3	3.4 ± 0.4	3.5 ± 0.3
P2-5	11.80	3.7 ± 0.1	5.3 ± 0.2
P2-10		3.2 ± 0.1	5.6 ± 0.1
P2-20		3.9 ± 0.1	5.1 ± 0.1
P2-25		4.2 ± 0.1	5.4 ± 0.0
P2-30		4.4 ± 0.1	5.0 ± 0.3
P2-40		4.3 ± 0.1	4.9 ± 0.1
P2-50		3.9 ± 0.2	3.9 ± 0.1
P2-80		2.6 ± 0.5	3.0 ± 0.2
P3-5	11.30	10.30	5.8 ± 0.1
P3-10			5.6 ± 0.3
P3-20			5.9 ± 0.4
P3-25			5.2 ± 0.2
P3-30			4.5 ± 0.4
P3-40			4.4 ± 0.3
P3-50			3.7 ± 0.2
P3-80			3.6 ± 0.2

Values are given in millivolts with SE. PIR, postinhibitory rebound.

The peak amplitudes of the three rebounds of LN5 elicited by one chirp clearly increased over the chirp when the test pulse was in the range of 5 to 20 ms (Fig. 3*B*, e.g., P1-5, P1-20), indicating an underlying enhanced depolarization of LN5. This tendency of the rebound to increase over a chirp became less prominent as the test pulse became longer in all three tests (e.g., P1-50, P1-80), which can be quantified by calculating the differences in amplitudes (slopes) of subsequent rebounds [Fig. 3*B*, P1-20, red arrows; see Zhang and Hedwig (18)]. For example, for the normal chirp *slope 1* is revealed from the resting potential at the start of the inhibition to the peak of the first rebound over the time between these two points, with a corresponding calculation for *slope 2* and *slope 3*. The overall trend of the change in the peak amplitude of the three PIRs was quantified by averaging the three slopes for each chirp (Fig. 3*D*). With increasing duration of the test pulse, the slope tuning curves in all three tests declined (P1- and P2-tests) or were flat (P3-test), and they did not match the phonotactic tuning for the P1-, P2-, and P3-tests.

**Figure 4.**
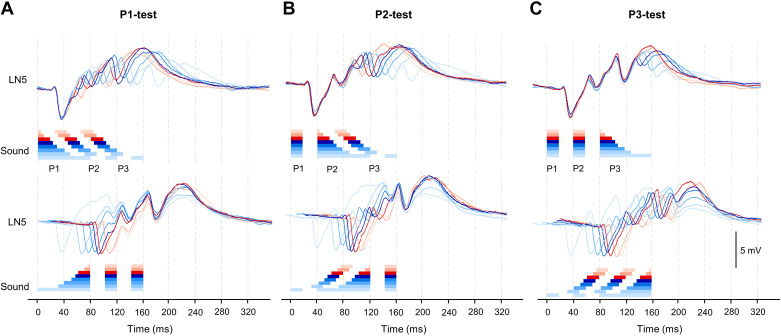
Averaged and superimposed LN5 membrane potential changes in response to the chirps of the P1-test (*A*), P2-test (*B*), and P3-test (*C*) patterns. *Top*: LN5 responses aligned to the start of the test pulse for each test pattern. *Bottom*: responses aligned to the end of the test pulse for each pattern. Short test pulses and responses are indicated by red colors and long pulses by light blue colors.

Aligning the LN5 activity elicited by each chirp reveals that in all three tests the first inhibition generated in LN5 was not affected regardless of which pulse within a chirp was varied (Fig. 4). The first inhibition always started with a latency of 26.4 ± 0.2 ms and reached an amplitude of −3.3 ± 0.0 mV with a latency of 36.4 ± 0.5 ms, which is 10.0 ± 0.1 ms after the onset of the inhibition, which supports the previous reports (12). In the P1-test, the start of the rebound depolarization leading to PIR1 is not coupled to the end of the sound pulses; rather it starts from the maximum of the initial inhibition. For all P1 pulses the first rebound initially follows a very similar time course (Fig. 4*A*, *bottom*). The amplitude of the inhibition elicited by the sound pulses was not affected by the change of the pulse duration and was similar across each test (Fig. 4).

#### Response of the coincidence detector neuron LN3.

**Figure 5.**
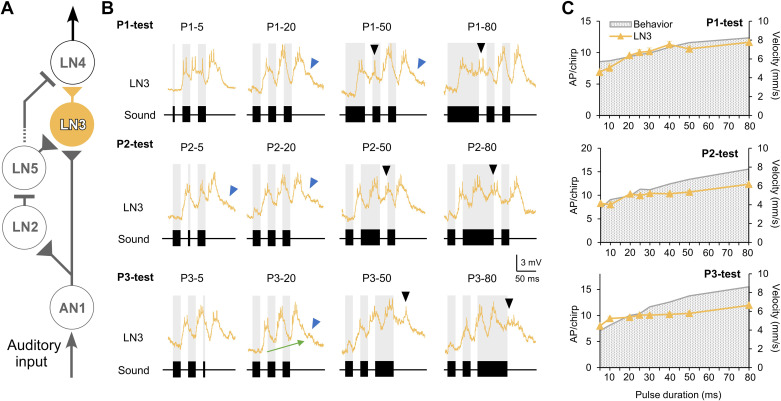
*A*: neural circuit proposed for the pattern recognition network with the coincidence detector neuron LN3 highlighted. *B*: intracellular recorded responses of LN3 to the P1-test (*top*), P2-test (*middle*), and P3-test (*bottom*) for 4 representative chirp patterns, respectively. Black arrowheads point to additional depolarization and spike bursts in response to long test pulses; blue arrowheads indicate graded responses likely elicited by the LN5 rebound. The green arrow in P3-20 indicates the increase of the depolarization along the processing of the chirp. *C*: tuning of the spiking response of LN3 (yellow) in comparison to the behavioral response (gray stippled background). AP, action potential.

Neuron LN3 functions as the coincidence detector in the network and is driven by a direct input from AN1 and a delayed graded input from LN5 (Fig. 5*A*). Based on the delay-line and coincidence detector mechanism, LN3’s response is enhanced after the second and the third pulse. When exposed to chirps with short test pulses, LN3 responded to the short pulse with a weak depolarization (Fig. 5*B*, P1-5). For chirps with medium pulses (e.g., P1-20), each pulse elicited a separate suprathreshold depolarization, in which the second and the third bursts of spikes were stronger than the first one (*P* < 0.0001, paired *t* test) (Table 4). Chirps with long pulses (e.g., P1-50 or P1-80) elicited a two-phase response with an initial burst of spikes followed by a weaker depolarization and spiking response (Fig. 5*B*, black arrowheads). When this additional excitatory activity occurred in response to long P1 or P2 pulses, it did not affect the activity elicited by subsequent pulses P2 or P3, respectively.

**Table 4.** Number of spikes elicited by each pulse of a chirp in LN3

Chirp	P1	P2	P3
P1-5	1.4 ± 0.4	2.0 ± 0.3	2.8 ± 0.7
P1-10	2.4 ± 0.4	2.4 ± 0.2	2.8 ± 0.7
P1-20	2.4 ± 0.2	3.4 ± 0.4	3.8 ± 0.2
P1-25	3.4 ± 0.4	3.6 ± 0.2	3.2 ± 0.3
P1-30	3.6 ± 0.4	3.6 ± 0.2	3.6 ± 0.2
P1-40	4.4 ± 0.4	3.8 ± 0.2	3.4 ± 0.2
P1-50	4.2 ± 0.4	3.6 ± 0.2	3.0 ± 0.0
P1-80	5.75 ± 0.2	3.0 ± 0.5	3.0 ± 0.0
P2-5	2.8 ± 0.2	1.8 ± 0.2	3.3 ± 0.2
P2-10	2.0 ± 0.0	3.0 ± 0.8	3.7 ± 0.3
P2-20	1.8 ± 0.2	3.8 ± 0.2	3.0 ± 0.6
P2-25	2.0 ± 0.5	3.3 ± 0.7	3.0 ± 0.5
P2-30	2.3 ± 0.2	3.5 ± 0.4	2.8 ± 0.4
P2-40	2.3 ± 0.2	3.0 ± 0.4	2.8 ± 0.6
P2-50	2.0 ± 0.0	4.7 ± 0.7	3.0 ± 0.0
P2-80	2.0 ± 0.0	5.3 ± 1.4	3.0 ± 0.0
P3-5	2.5 ± 0.3	4.0 ± 0.0	1.5 ± 0.3
P3-10	2.5 ± 0.4	4.5 ± 0.3	3.3 ± 0.2
P3-20	2.5 ± 0.4	4.3 ± 0.2	4.0 ± 0.0
P3-25	2.5 ± 0.2	4.0 ± 0.0	4.0 ± 0.0
P3-30	2.25 ± 0.2	4.0 ± 0.0	4.2 ± 0.0
P3-40	2.5 ± 0.4	4.5 ± 0.4	3.8 ± 0.2
P3-50	2.25 ± 0.5	3.8 ± 0.2	4.8 ± 0.2
P3-80	2.0 ± 0.4	4.0 ± 0.0	5.8 ± 0.6

Values are given in action potentials (AP)/pulse with SE.

An excitatory postsynaptic potential (EPSP) could occur after the response to a chirp with a latency of 68.6 ± 0.9 ms to P3 (Fig. 5*B*, blue arrowheads). Both the additional depolarization (Fig. 5*B*, black arrowheads) and the EPSP at the end (Fig. 5*B*, blue arrowheads) may be linked to the PIRs of LN5, which are an input to LN3. Over the time course of a chirp, the membrane potential of LN3 did not completely repolarize in the pulse intervals; rather an overall graded depolarization of LN3 occurred for normal chirps (Fig. 5*B*, P3-20, green arrow).

Quantitative analysis revealed that the LN3 activity showed significant differences with increasing duration of the test pulse in all three tests (*P* < 0.0001, Friedman test, *N* = 5, *n* = 13). The number of spikes elicited by each chirp increased from 6.9 ± 0.6 to 11.6 ± 0.5 APs/chirp in the P1-test, from 8.3 ± 0.3 to 12.4 ± 0.4 APs/chirp in the P2-test, and from 8.0 ± 0.3 to 12.0 ± 0.6 APs/chirp in the P3-test. The tuning curves of LN3 in all three tests showed overall a rising tendency with the increase in test pulse duration. For example, as P1 increased from 5 ms to 40 ms, LN3 activity linearly increased from 6.9 ± 0.6 to 11.3 ± 0.5 APs/chirp (P1-5 vs. P1-20: *P* = 0.0428, Friedman test, *N* = 5, *n* = 13). Its activity then stayed at a similar level, giving 10.6 ± 0.4 APs/chirp at P1-50 and 11.6 ± 0.5 APs/chirp at P1-80 (P1-80 vs. P1-20: *P* = 0.02, Friedman test, *N* = 5, *n* = 13). Thus, the LN3 response to the P1-test matched the behavioral tuning (Fig. 5*C*). The LN3 tuning curves for the P2- and P3-tests increased less with increasing pulse duration, and for longer test pulses these tuning curves showed a weaker match to the behavior.

#### Response of the feature detector neuron LN4.

**Figure 6.**
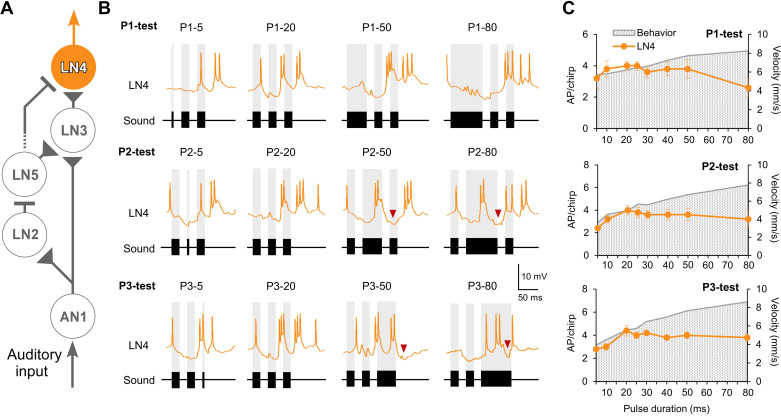
*A*: neural circuit proposed for the pattern recognition network with the feature detector neuron LN4 highlighted. *B*: intracellular recorded responses of LN4 to the P1-test (*top*), P2-test (*middle*), and P3-test (*bottom*) for 4 representative chirp patterns, respectively. Red arrowheads point to an inhibition following the short membrane depolarization and burst of spikes in response to long test sound pulses. *C*: tuning of the spiking response of LN4 (orange) in comparison to the behavioral response (gray stippled background). AP, action potential.

The activity of the feature detector LN4 is driven by an inhibitory input from LN2 and an excitatory input from LN3 (Fig. 6*A*). In response to the P1-5 chirp, LN4 generated a very weak inhibition following the 5-ms pulse; the subsequent 20-ms pulse elicited 0.8 ± 0.4 APs (Fig. 6*B*, P1-5). When the duration of the first sound pulse in the P1-test increased to P1-80 ms, the inhibition more and more dominated the initial response of LN4: it occurred with a latency of 25.1 ± 0.6 ms and was mixed with subthreshold EPSPs. The second and the third pulse each elicited a pronounced suprathreshold depolarization with bursts of 2 or 3 spikes (e.g., Fig. 6*B*, P1-50). The burst of spikes in response to the second sound pulse was always strong, whereas spiking activity in response to the third pulse did not generate a clear burst for P1-50 and P1-80. In the P2-test the response of the varied test pulse interacted with the initial inhibition, triggered by a constant 20-ms pulse. The short pulse P2-5 elicited only 0.6 ± 0.2 APs (Fig. 6*B*, P2-5), whereas 2 bursts of spikes occurred for the P2-20 chirp. As the duration of the P2 test pulse exceeded 20 ms, a mixed excitatory-inhibitory response occurred with a burst of spikes preceded and followed by an inhibition (Fig. 6*B*, P2-50 and P2-80, red arrowheads). As a consequence of the interaction of excitation and inhibition, the depolarization in response to the P2-50 and P2-80 pulse was cut short to 33.7 ± 0.7 ms, and it was outlasted by the sound pulse. The inhibitory activity did not affect the excitatory response to the third pulse of the P2 chirps, giving 2.0 ± 0.1 APs/pulse in every chirp (Table 5). Also, in the P3-test long test pulses only elicited a short excitatory response with a burst of spikes on top of a depolarization lasting for 31.76 ± 0.8 ms terminated by a subsequent inhibition (Fig. 6*B*, P3-50, P3-80, red arrowheads).

**Table 5.** Number of spikes elicited by each pulse of a chirp in LN4

Chirp	P2	P3
P1-5	0.8 ± 0.4	2.4 ± 0.2
P1-10	1.4 ± 0.4	2.4 ± 0.2
P1-20	1.6 ± 0.2	2.4 ± 0.2
P1-25	2.0 ± 0.3	2.0 ± 0.0
P1-30	1.4 ± 0.2	2.2 ± 0.2
P1-40	1.6 ± 0.2	2.2 ± 0.3
P1-50	2.0 ± 0.4	1.8 ± 0.3
P1-80	1.6 ± 0.4	1.0 ± 0.3
P2-5	0.6 ± 0.2	1.8 ± 0.3
P2-10	0.8 ± 0.3	2.4 ± 0.2
P2-20	1.8 ± 0.3	2.2 ± 0.2
P2-25	1.6 ± 0.2	2.2 ± 0.3
P2-30	1.8 ± 0.2	1.8 ± 0.3
P2-40	1.6 ± 0.2	2.0 ± 0.4
P2-50	1.8 ± 0.2	1.8 ± 0.4
P2-80	1.8 ± 0.3	1.4 ± 0.6
P3-5	1.6 ± 0.2	1.2 ± 0.2
P3-10	1.6 ± 0.2	1.4 ± 0.2
P3-20	2.0 ± 0.3	2.4 ± 0.2
P3-25	2.0 ± 0.3	2.0 ± 0.0
P3-30	2.0 ± 0.0	2.2 ± 0.2
P3-40	1.8 ± 0.2	2.0 ± 0.0
P3-50	1.8 ± 0.2	2.2 ± 0.2
P3-80	1.8 ± 0.3	2.0 ± 0.4

Values are given in action potentials (AP)/pulse with SE.

The tuning curves of the LN4 responses to the three sequences of test patterns showed a similar trend (Fig. 6*C* and Table 1). An increase when the duration of the test pulse increased to 20 ms was followed by a gradual decline. The activity elicited by chirps with a 5-ms test pulse (P1-5, P2-5, and P3-5: 2.2 ± 0.2 APs; 2.2 ± 0.3 APs; 2.4 ± 0.2 APs) was always lower than the activity in response to a normal chirp (all pulses 20 ms long: 3.8 ± 0.2 APs; 3.8 ± 0.3 APs; 4.4 ± 0.5 APs) and declined to chirps with long test pulses (P1-80, P2-80, and P3-80: 3.2 ± 0.4 APs; 3.4 ± 0.8 APs; 3.6 ± 0.2 APs). In all three tests, the tuning curve of LN4 matched the phonotactic tuning when the test pulse was equal to or shorter than 20 ms but declined for patterns with longer sound pulses.

#### Pattern-specific processing in LN3 and in LN4.

**Figure 7.**
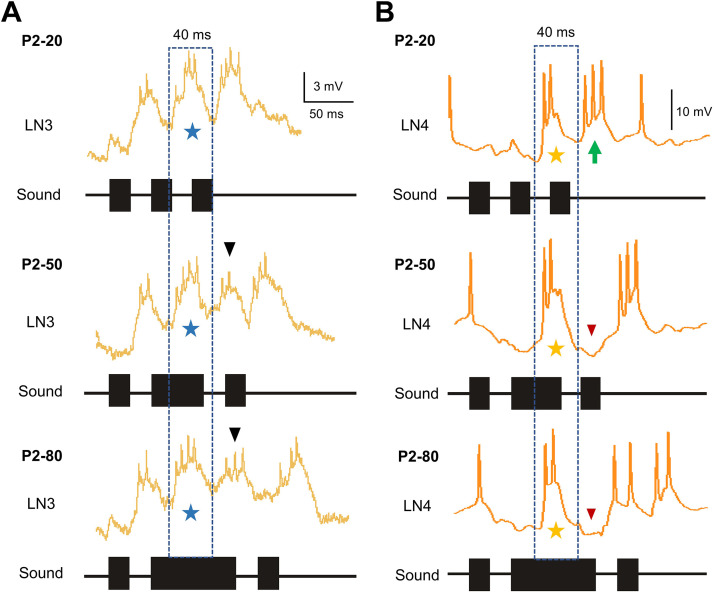
*A*: response of LN3 to long test pulses of the P2-test pattern. The typical response to a 20-ms pulse (*top*) occurs also for a 50-ms and a 80-ms test pulse (*middle* and *bottom*; blue stippled box and stars). Long test pulses initiate an additional depolarizing LN3 response, which is not coupled to the temporal pattern of the sound (black arrowheads). *B*: response of LN4 to pulses of the P2-test pattern. The depolarizing response to a 20-ms pulse (*top*) also occurs for a 50-ms and a 80-ms pulse (*middle* and *bottom*; blue stippled box and stars). The long pulses, however, also elicit an inhibition, which follows and terminates the initial depolarizing response (red arrowheads). The green arrow indicates the last burst of spikes elicited by the third sound pulse.

Two features of the LN3 and LN4 responses over the time course of long test pulses stand out: the extra depolarization occurring in LN3 and the shortened depolarizing response revealed in LN4 (Fig. 7). When P2, i.e., the second sound pulse of a chirp, was 50 or 80 ms, LN3 responded with an initial burst of spikes (Fig. 7*A*, blue stars) followed by an additional depolarization (Fig. 7*A*, black arrowheads). The initial depolarization triggered by a long P2 test pulse had a duration of 38.7 ± 0.5 ms regardless of the duration of the pulse, and the concomitant burst of spikes (3.8 ± 0.3) lasted for 18.6 ± 0.3 ms. The subsequent additional depolarization occurred 65.3 ± 0.3 ms after the onset of the test pulse and ∼40 ms after the start of the initial depolarization, corresponding to the moment when in response to a normal chirp the third burst of spikes will start (compare with P2-20). So, for long P2 test pulses LN3 activity did not follow the duration of the sound, but its membrane potential rather oscillated like responding to a normal chirp pattern.

In LN4, P2 test pulses triggered a pronounced depolarization with a burst of spikes (Fig. 7*B*, yellow stars). This was followed by an inhibition that became more prominent as the duration of P2 increased (Fig. 7*B*, red arrowheads). The 50- to 80-ms duration of the test pulse was not matched by a corresponding long depolarization of LN4. Because of the subsequent inhibition, the depolarization elicited by these long pulses was cut short to 37.8 ± 0.8 ms (Table 5). This is very similar to the duration of the initial depolarization in response to long P2 pulses described in LN3 (Fig. 7*A*, blue stars). The restricted excitatory response of LN4 to a long P2 test pulse appears to occur during an ongoing inhibition; it matches the depolarization elicited by a normal 20-ms pulse (compare to P2-20). This implicates that the effective sound pulse duration processed in the circuit may be limited by an ongoing inhibition initiated by long sound pulses. Note that in response to the normal chirp (P2-20) the last burst of spikes triggered by the third sound pulse P3 (Fig. 7*B*, green arrow) starts right at the time when in response to P2-50 and P2-80 the inhibition occurred (red arrowheads, compare top and bottom recordings). This may indicate a form of “timed inhibition” that corresponds to the conspecific temporal pattern.

Both neurons also showed corresponding response patterns in the P3-test. In LN3, the initial depolarization elicited by a long P3 test pulse lasted 39.9 ± 0.6 ms and the concomitant spiking for 19.3 ± 1.0 ms. A subsequent depolarization occurred with a latency of 74.7 ± 1.3 ms to the onset of the P3 test pulse. For LN4, the depolarization in response to a 50- or 80-ms P3 test pulse was 36.4 ± 1.0 ms and shorter than the duration of the sound pulses. This cutoff effect of LN4 activity again indicates a filtering mechanism in the network for pulse duration, which may depend on the balanced input of excitation via LN3 and inhibition via LN2.

## DISCUSSION

**Figure 8.**
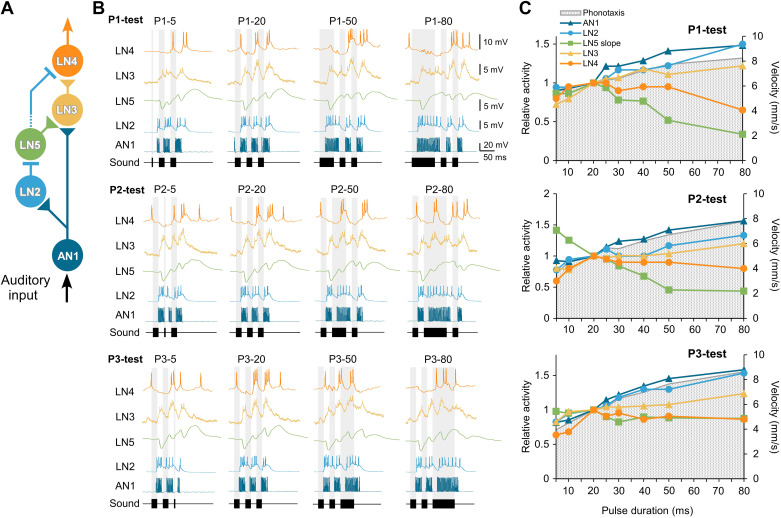
*A*: neural circuit proposed for the pattern recognition network with all neurons addressed. *B*: comparative display with recordings of all 5 neurons tested with the P1-test, P2-test, and P3-test patterns; recordings obtained in different animals. *C*: comparison of the neuronal tuning curves with the behavioral tuning to the different test patterns.

We analyzed the response patterns of local auditory brain neurons to changes in the duration of sound pulses and used three different chirp paradigms to further elucidate underlying cellular and network mechanisms of cricket song pattern recognition, which has been studied as a model for temporal processing (10, 26). The recordings and behavioral tuning curves are summarized in Fig. 8.

### Experimental Considerations: Modulation of Phonotactic Behavior

To allow sufficient time for testing neural responses during intracellular recordings, in the present experiments the chirps of the test paradigms were presented in a looped mode. In comparison to previous phonotaxis experiments, where each type of chirp was played for 60 s (19), the tuning of the behavior was considerably broader and showed pronounced responses to chirps with long test pulses. Previous experiments demonstrated that long nonattractive sound pulses elicit a strong steering response when inserted into a sequence of normal chirps (20, 21). In each loop at least three attractive normal chirps occurred where the altered pulse was between 20 and 30 ms. In line with the previous experiments, we think that these chirps had a modulatory effect leading to the pronounced steering response to longer pulses as reflected in the behavioral tuning curves. Please note that the behavior is different and narrowly tuned if all pulses of a chirp have the same duration and if the patterns are not presented in a looped mode (8, 9, 17).

### Response Properties of Neurons in the Pattern Recognition Network

The components of the proposed pattern recognition network in the brain of *G. bimaculatus* (Fig. 8*A*) are the axon terminals of the ascending neuron AN1 and locally a delay line (LN2 and LN5), a coincidence detector (LN3), and a final feature detector neuron (LN4) (10, 12, 17). The network shares fundamental features of pulse pattern processing as elucidated in other insects and in fish, frogs, and higher vertebrates.

#### Ascending neuron AN1 and local neuron LN2.

Neurons AN1 and LN2 copy the pulse pattern in their spike activity (17, 18, 26). In our experiments spike activity of AN1 and LN2 closely matched the tuning of the behavior, and their activity increased with pulse duration. This correlation, however, should not be interpreted as an indication of pattern recognition at the level of these neurons (see below), as the spike activity of the ascending AN1 just mirrors the pulse duration.

In comparison to AN1, spike activity of the local neuron LN2 was considerably lower (Fig. 8*B*), it always showed a pronounced phasic onset response to the first pulse of a chirp, and its depolarization always outlasted the pulse duration and led to a maintained depolarization over the time course of the chirps (17). As LN2 forwards inhibition to LN5 and LN4 (12), its activity will shape the response patterns of these neurons.

#### Nonspiking delay-line neuron LN5.

The characteristic feature of the LN5 neuron is an initial inhibitory response to sound pulses followed by a subsequent postinhibitory rebound (Fig. 8*B*) (12, 18). Such a combination of inhibition and postinhibitory rebound is an essential mechanism for processing sequences of sound pulses in fish (13, 14), frogs (27), echo-delay-sensitive neurons in bats (15), and for sound pulse duration tuning of neurons in bats (16, 28), in mice (29), and likely across vertebrates (30). It may be regarded as a fundamental processing mechanism in neural systems. Although the underlying currents driving the rebound dynamics have been identified in some vertebrates, a characterization of conductances in LN5 is not yet available but might be crucial to understand the tuning of species-specific recognition processes (18). Support for the processing of long pulse intervals in the insect brain by inhibition comes from the bush cricket *Ancistrura nigrovittata*. In a local auditory brain neuron, the timing of a long-lasting inhibition in response to a chirp appears well adapted to the species-specific communication, although in this case it lacks a rebound (31).

When the responses to altered pulse intervals were considered (18), the rebound activity of the nonspiking interneuron LN5 showed a close correlation to the behavioral tuning. In the pulse duration paradigm tested here such a close link between LN5 activity and the behavioral tuning is not obvious (Fig. 8*C*). With increasing duration of the first (P1) or second (P2) test pulse in the chirps, the rebound activity of LN5 became distorted because of additional rebound peaks: it overall decreased in amplitude and did not follow the behavioral tuning. The origin of the additional rebound responses is not obvious. They cannot be explained by the activity of its only known input, the LN2, and could indicate additional inhibitory inputs to LN5 driven by rhythmic network activity (see below).

At the cellular level the systematic variation of the sound pulse duration (Fig. 4) reveals that after a sound pulse the rebound process in LN5 always starts with a highly similar time course and amplitude after ∼26.4 ± 0.2 ms. Depending on the pulse duration, it subsequently reached different rebound amplitudes after the end of the sound pulse. The rebound is terminated by the subsequent inhibition, or it just decays after the last pulse of a chirp. Therefore, the start of the rebound is triggered by a stereotyped inhibition following a sound pulse of a chirp, whereas its amplitude and time course are related to the duration of the sound pulses. The onset of LN5 inhibition at the start of a chirp closely matches the onset of phasic LN2 spike activity.

The recordings also revealed a consistent increase in the LN5 membrane potential and an increase in the amplitude of the rebounds over the time course of a normal chirp (Fig. 8*B*) (18), similar to the increasing responses of “interval-counting” neurons in the torus semicircularis in frogs (32). It may be linked to a declining inhibitory impact of LN2 over a chirp and support the processing of chirp durations, as for song pattern recognition an additional filter for the timing of chirps may operate (33). As a nonspiking neuron, LN5 will drive activity of postsynaptic neurons by a graded transmitter release coupled to its membrane potential (34), and its gradually increasing membrane potential together with the rebound potentials will contribute to the activity of the coincidence detector LN3.

#### Coincidence detector neuron LN3.

Coincidence detection is best known for the processing of dichotic signals in vertebrates with timing differences in the submillisecond to millisecond range (35–37). For processing of acoustic communication signals like in crickets or fish (13), it is based on a delayed rebound excitation to an initial sound pulse P1 coinciding with the excitatory response to a subsequent pulse P2. The timing of the inhibition and rebound matches the period of the sound pulses as an essential part of the pattern recognition process (12, 18). A corresponding mechanism for sound pulses likely operates in fish (14), in frogs (27), and in bats for pulse-echo delays of several milliseconds. Neurons representing target range are tuned by a combination of delay by lasting inhibition, rebound, and coincidence detection coupled to a sonar pulse and its echo (15, 38).

The activity of the coincidence detector neuron LN3 increased with increasing pulse duration and at least for the P1-test pattern showed a good match to the behavior (Fig. 8, *B* and *C*). Its activity is driven by coincident spiking activity of AN1 with a graded input from LN5. Based on the timing of AN1 spike activity and the LN5 postinhibitory rebound, both overlapped in response to the second (P2) and third (P3) pulses of a chirp for ∼7 ms before the rebound was terminated by a subsequent inhibition. This allowed ∼2–3 spikes of AN1 to coincide with the postinhibitory rebound and to boost LN3 activity (11, 17). The membrane potential of LN3 also revealed an increase over the duration of a chirp, in line with the increase of the LN5 membrane potential and its rebound responses.

With long constant pulses, additional bursts of activity occurred in LN3. A rhythmic response to a constant long sound pulse is also evident in previous LN3 recordings (see Fig. 3B in Ref. 17) providing evidence for resonant-like properties, either intrinsic to LN3 and/or to the network as described for pattern recognition in a bush cricket (39, 40). As additional rhythmic activity also occurred in LN5 and in LN4 (see below) a network property leading to the oscillation seems more likely. Interestingly, the rhythmic activity of LN3 is not reflected in the spiking response of LN4 (see below).

#### Feature detector neuron LN4.

Activity of neuron LN4 closely matches the tuning of phonotactic behavior to systematic changes in pulse periods (12, 17). The neuron may represent the highest level of processing in the song pattern recognition network and has been indicated as the system’s feature detector (41, 42), specifically representing the presence of the calling song pulse pattern. Spike activity in LN4 is considerably lower than activity of the first-order interneuron AN1 (12, 17, 18). Such a sparse coding like in other sensory systems points toward a higher relevance of LN4 activity and to a semantic shift in coding of the sound signals as described for auditory neurons in locusts (43) and in the inferior colliculus of cat (44).

The response of the feature detector neuron LN4 is shaped by an interplay of inhibition via LN2 and excitation via LN3 (Fig. 8*B*), and like in frog interval-counting neurons (27) this interaction provides the basis for its pulse interval-selective responses (12, 17, 18). When the duration of a sound pulse P1 increased, the response of LN4 showed a strong inhibition, in line with a corresponding extended LN2 activity. When 50- to 80-ms-long P2 or P3 were inserted in a chirp, LN4 gave a surprisingly short excitatory response to the start of the pulse (Figs. 6 and 7), which occurred embedded in an ongoing inhibition corresponding to models for duration-selective neurons in frogs (27). Inhibition in LN4 matched the spike activity of LN2 (Figs. 2 and 7). A very similar short response of LN4 to long sound pulses due to an interplay of inhibition and excitation has been reported before (10). Within the context of the tests performed here, it indicates a selective sound pulse duration detector, which shapes the response of the LN4 neurons to the pulse duration of the species-specific song pattern. Such a pulse duration filter would be well adapted for processing the stereotyped pattern of the cricket’s calling song and would tune the pattern recognition network in complex acoustic situations to the pattern that matches best. Therefore, in these experiments, different from the neurons at the earlier stages of the network, LN4 spike activity declines with increasing pulse durations and does not follow the tuning of the behavior (Fig. 8*C*); rather it shows a tuning matching the behavioral tuning to different pulse periods. This “pulse detection” property of LN4 is a new feature in the system, which complements delay-line and coincidence detection and requires further attention, like intracellular excitatory or inhibitory stimulation to test whether LN4 neurons can initiate, suppress, or modulate phonotactic behavior.

### Modulation of the Phonotactic Response

In the present experiments spike activity of LN4 is well aligned with the behavioral response to chirps with varied sound pulses shorter than the normal pulses but does not match the behavioral response to chirps with longer varied pulses (Fig. 6). This indicates an apparent discrepancy between the tuning of behavior and the feature detector neuron, which did not occur when changes in pulse intervals were analyzed (18). From a conceptual point of view, pattern recognition and steering might be organized in a parallel or a serial way (45); however, the functional organization is more dynamic. Nonattractive long sound pulses drive pronounced auditory steering responses in behavioral experiments when combined with the normal pulse pattern that initiates the recognition process (21). This has been described as tolerant pattern recognition (20). The modulation of the phonotactic response could either occur at the level of the recognition process or at the level of the auditory steering process (21). There is no evidence for an enhanced response to long pulses by the feature detector neuron LN4, regarded as the output stage of the pattern recognition network (12); and rather the opposite occurs. Phonotactic steering may be organized at the thoracic level responding to auditory activity as shown by AN1, which is driven by auditory afferents (46). Our physiological evidence indicates that, once pattern recognition is established, the steering to unattractive sound pulses bypasses the level of pattern recognition, although the brain could still play a role via the short-latency auditory-responsive descending brain neuron that also copies the sound pattern (47). Such a parallel organization of pattern recognition and steering is also indicated by behavioral experiments in crickets (23, 48, 49) and grasshoppers (45).

## DATA AVAILABILITY

Data will be made available upon reasonable request.

## SUPPLEMENTAL MATERIAL

10.6084/m9.figshare.24131553 Supplemental Table S1: https://doi.org/10.6084/m9.figshare.24131553.

## GRANTS

X.Z. was funded by the Cambridge Trust, the Department of Zoology, and a Trinity-Henry-Barlow scholarship and by Biotechnology and Biological Sciences Research Council (BBSRC) Grant BB/T002085/1. Equipment used was funded by various BBSRC grants to B.H.

## DISCLOSURES

No conflicts of interest, financial or otherwise, are declared by the authors.

## AUTHOR CONTRIBUTIONS

X.Z. and B.H. conceived and designed research; X.Z. performed experiments; X.Z. analyzed data; X.Z. and B.H. interpreted results of experiments; X.Z. prepared figures; X.Z. drafted manuscript; X.Z. and B.H. edited and revised manuscript; X.Z. and B.H. approved final version of manuscript.
